# Endoscopic treatment for a giant gastric bezoar: Sequential use of electrohydraulic lithotripsy, alligator forceps, and snares

**DOI:** 10.1002/jgh3.12491

**Published:** 2021-01-27

**Authors:** Ryuhei Jinushi, Takahiko Yano, Noriatsu Imamura, Naoki Ishii

**Affiliations:** ^1^ Gastroenterology Center Tokyo Shinagawa Hospital Tokyo Japan

**Keywords:** electrohydraulic lithotripsy, endoscopic treatment, gastric bezoar

## Abstract

A 62‐year‐old woman with no past history was referred to our hospital for endoscopic treatment of a large gastric bezoar measuring 10 cm in diameter. The bezoar had a hard surface and huge volume. A tunnel was created at the center of the bezoar using electrohydraulic lithotripsy and was dilated using a through‐the‐scope balloon. The bezoar was then gradually crushed using alligator forceps and snares to decrease the risk of intestinal obstruction by the crushed bezoar fragments. The sequential use of electrohydraulic lithotripsy, alligator forceps, and snares according to the therapeutic plan enabled the endoscopic treatment of the giant gastric bezoar without surgery.

## Graphical abstract

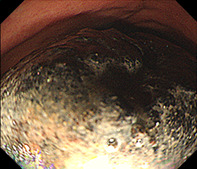

A bezoar measuring 10 cm in diameter was found to have a hard surface and huge volume. The sequential use of electrohydraulic lithotripsy, alligator forceps, and snares according to the therapeutic plan enabled the endoscopic treatment of the giant gastric bezoar without surgery.

## Introduction

Gastric bezoars are very rare entities, and the proportion of gastric bezoars among subjects who underwent esophagogastroduodenoscopy was 0.3%, although the actual rate is considered quite lower due to the current widespread use of endoscopy.^1^ The decrease in gastric motility was considered one of important risk factors for the development of gastric bezoars.^2^ Recent advancements in endoscopic modalities have enabled the application of several endoscopic therapies for the treatment of gastric bezoars.^3^, ^4^, ^5^, ^6^, ^7^ Although there are no established methods for the treatment of gastric bezoars, there are reports of endoscopic treatment using dual knife, electric snare, and guidewire. Adverse events after endoscopic therapy, such as intestinal obstruction caused by residual bezoars, have also been reported. Therefore, careful strategic planning is required to prevent adverse events.^6^ Here, we report our experience of endoscopically treating a giant gastric bezoar with a variety of instruments without complications.

## Case report

**Figure 1:**
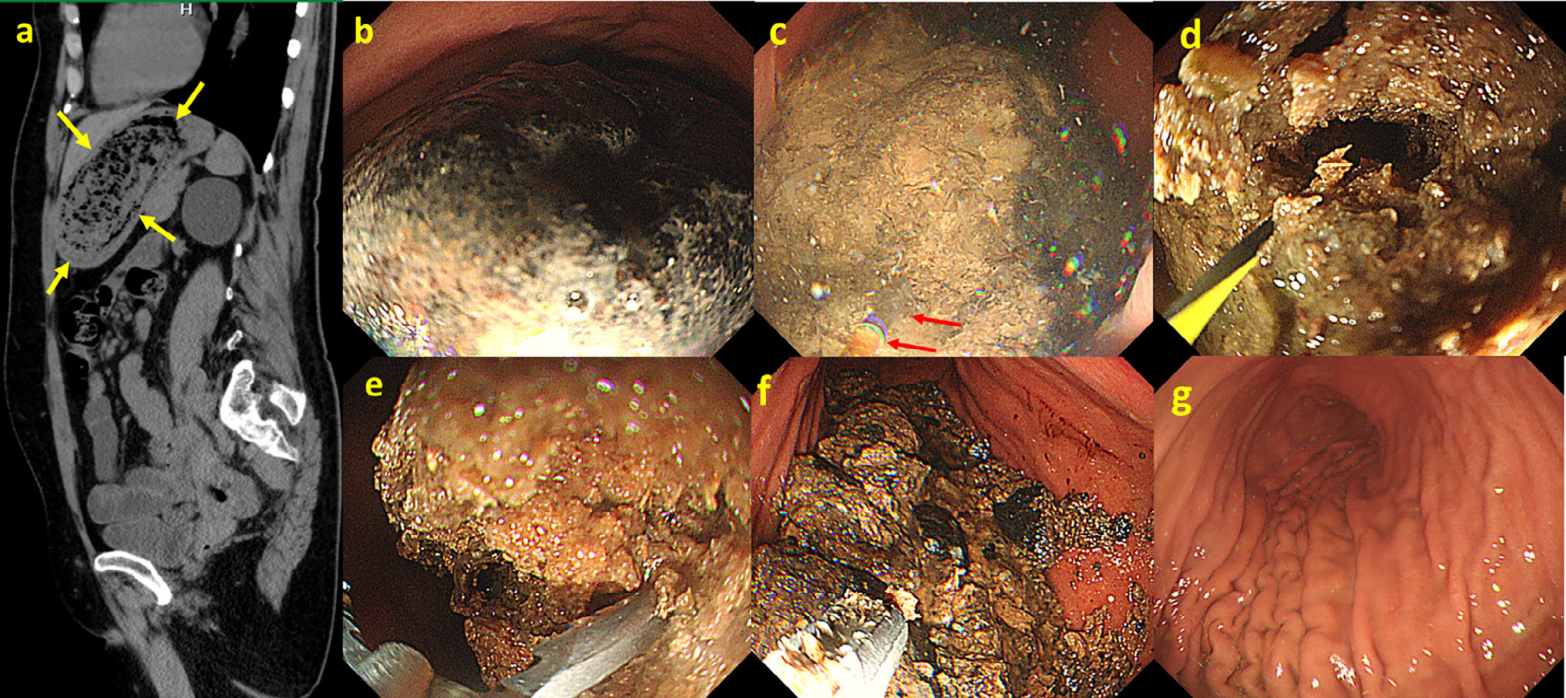
(a) Sagittal computed tomography image showing a large gastric bezoar occupying the gastric body and fundus (➨). The bezoar measures 10 cm along its longest diameter. (b) Endoscopic view of a giant gastric bezoar with a blackish surface and hard texture. (c) Electrohydraulic lithotripsy is performed to create a tunnel at the center of the bezoar (➨). (d) Balloon dilation is performed after electrohydraulic lithotripsy to enlarge the tunnel. (e) The bezoar is grasped using alligator forceps and gradually crushed. (f) The bezoar is crushed using alligator forceps and snares. (g) The giant bezoar is completely crushed and removed without complications. Large fragments of the bezoar are retrieved, and small fragments are allowed to pass into the intestine.

A 62‐year‐old woman with no past history was referred to our hospital for the endoscopic treatment of a large gastric bezoar measuring 10 cm in diameter (Fig. 1a). Physical examination showed intermittent upper abdominal pain, but no other obvious abnormalities were found. Laboratory tests revealed dyslipidemia (triglyceride 189 mg/dL, total cholesterol 273 mg/dL, and low‐density lipoprotein cholesterol 174 mg/dL). No anemia (red blood cells 462 × 10^4^/L, hemoglobin level 13.4 g/dL) was observed. Serum albumin (4.2 g/dL), total bilirubin (0.61 mg/dL), aspartate aminotransferase (17 U/L), alanine aminotransferase (13 U/L), alkaline phosphatase (43 U/L), and creatinine levels (0.75 mg/dL) were within the normal limits. Regarding her drug history, she had been taking over‐the‐counter supplements containing indigestible dextrin for several months. At the previous hospital, endoscopic treatment with mechanical lithotripters and dissolution therapy using Coca Cola was unsuccessful. Because endoscopic therapy is less invasive compared to surgery, we also selected endoscopic fragmentation as the first treatment option in the inpatient setting. However, the bezoar could not be grasped using endoscopic lithotripters or snares because of its huge volume and hard surface (Fig. 1b). Therefore, a tunnel was created at the center of the bezoar using electrohydraulic lithotripsy (760 mJ/5 Hz, Lithotron EL27 Compact, Walz Elektronik GmbH, Rohrdorf, Germany) (Fig. 1c), and a guidewire (Jagwire 0.0035 inch, Boston Scientific Corp, Marlborough, MA, USA) was passed through this tunnel. Subsequently, the tunnel was dilated using a through‐the‐scope balloon (Hurricane TX, 8 mm in diameter and 4 cm in length; Boston Scientific Corp) (Fig. 1d). The bezoar could then be grasped using alligator forceps and was gradually crushed to decrease the risk of intestinal obstruction by the crushed bezoar fragments (Fig. 1e). Snares were also used during the process. Approximately half of the treatment process was completed on the first day (Fig. 1f). As the residual bezoar was still large in size, the risk of the bezoar passing into the intestine was considered low. Two additional treatment sessions were performed within 1 week, and the total treatment time was about 4 h. The residual bezoar was split into fragments, and efforts were made to collect as many fragments as possible. When collection was difficult, the fragments were further split into smaller fragments measuring <2 cm. Follow‐up esophagogastroduodenoscopy was performed 2 weeks after the initial endoscopic treatment, and the patient was discharged without complications, with no residual bezoars present in the stomach (Fig. 1g). Later, a compositional analysis of the gastric bezoar was performed, but no significant results were obtained. However, the supplement she had been taking for a long time was likely to be the cause.

## Discussion

Surgical treatment is generally chosen for giant gastric bezoars owing to the technical difficulties associated with the endoscopic treatment. Reports of gastric bezoars treated using endoscopy have been published, and grasping a gastric bezoar is considered the first step of the endoscopic treatment for bezoars.^3^, ^4^, ^5^, ^6^, ^7^, ^8^ In the present case, owing to the hard surface and huge volume of the gastric bezoar, which measured 10 cm in diameter, the bezoar could not be grasped using lithotripters or snares, and holes or grooves on the surface of the gastric bezoar were required. Therefore, electrohydraulic lithotripsy, which has been used for the treatment of refractory biliary stones,^9^ was first applied to the creation of the hole before using alligator forceps and snares, unlike in the previous reported cases. After dilating the hole using a through‐the‐scope balloon, the bezoar could be grasped and crushed using alligator forceps. The second important step was to grasp the snares. The volume and diameter of the huge bezoar diminished using the alligator forceps, and the bezoar could be grasped with a snare. Endoscopic fragmentation was performed using the snare and alligator device. The third considerable point was to crush the huge bezoar to pieces to prevent small bowel obstruction after parts of the crushed bezoar flowed into the small intestine.^6^ The combined use of alligator forceps and snares was also effective in crushing the large bezoar to pieces to prevent small bowel obstruction caused by residual bezoars. As a result, the huge gastric bezoar could be treated endoscopically without complications, and surgery could be obviated. However, the recurrence rate of gastric bezoars after treatment was reported to be 20% in the previous report.^10^ Increasing the intake of water, modifying diet, and chewing food sufficiently may be ways to avoid recurrence. The sequence use of endoscopic modalities according to the therapeutic plan enabled the endoscopic treatment of a giant gastric bezoar without surgery.

## Supporting information


**Video S1.** Supplementary video.
